# Two Cases of Multiple Thromboembolism With Asymptomatic Atrial Fibrillation

**DOI:** 10.7759/cureus.21645

**Published:** 2022-01-26

**Authors:** Yudai Tanaka, Michiaki Matsumoto, Takaharu Yahata, Takashi Mineki, Koji Oiwa

**Affiliations:** 1 Cardiology, Yokohama Chuo Hospital, Yokohama, JPN; 2 Cardiology, Nihon University School of Medicine, Tokyo, JPN

**Keywords:** anticoagulant therapy, severe complication, multiple thromboembolism, asymptomatic patient, atrial fibrillation

## Abstract

A 70-year-old woman was brought to our hospital by ambulance due to syncope and hemiparalysis. Brain magnetic resonance imaging (MRI) and an electrocardiogram (ECG) showed the cerebral infarction and ST elevation without chest pain. The neurological findings were improved at the emergency outpatient service, therefore an emergency coronary angiography and aspiration for the thrombus was performed for acute myocardial infarction. An electrocardiogram monitor revealed asymptomatic and paroxysmal atrial fibrillation (AF) on the third day. In the other case, an 88-year-old woman was admitted to ambulatory care for abdominal pain, and the abdominal ultrasound showed findings of splenic infarction. Although there were no chest symptoms, AF was observed on the electrocardiogram at the time of admission. And endoscopic ultrasonography and brain MRI during hospitalization showed splenic infarction and multiple infarctions. Here, we report two cases with multiple thromboembolic complications associated with asymptomatic AF.

## Introduction

Although it is well-known that the thromboembolism caused by atrial fibrillation (AF) is serious and prevention is important, little is known about the existence of undetected asymptomatic AF patients. The prevalence rate of AF in the elderly has been reported to be 4.3% to 14% in their 70s and 9% to 13% in their 80s [1-3], and another report has shown the incident rate of asymptomatic AF events is 3.7%/year in Japanese [4], suggesting that there are more undetected asymptomatic AF patients in the elderly. There are several reports on the prediction and method of detecting AF, the CHA2DS2-VASc score (congestive heart failure, hypertension, age ≥75 (doubled), diabetes, stroke (doubled), vascular disease, age 65 to 74 and sex category (female)) and HAS-BLED score are significantly higher in asymptomatic AF patients [5], and no history of β-blocker administration and a high serum level of NT-proBNP are independent predictors for the findings of undetected AF [4], however, those are not enough to provide a reliable method for the detection of AF. We also might need to educate the home pulse check or recommend the use of cardiac monitor to those patients. Here, we show two cases of multiple thromboembolic diseases caused by AF, which were accidentally found during admission, suggesting that an additional study for the early detection of AF is needed to prevent thromboembolic complications.

## Case presentation

Case 1

**Figure 1:**
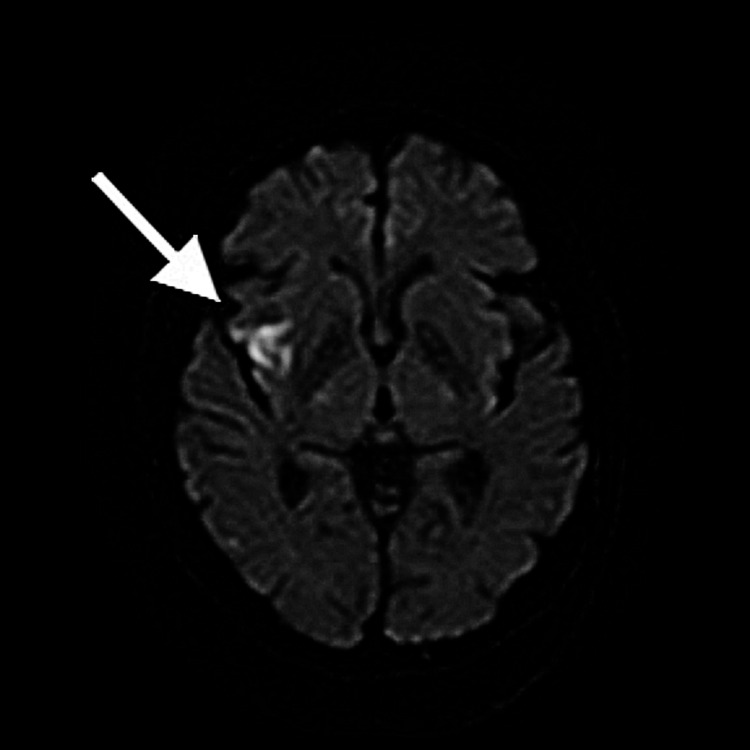
Brain MRI image Brain MRI on admission in Case 1 shows a new infarction in the upper right cortex

**Figure 2:**
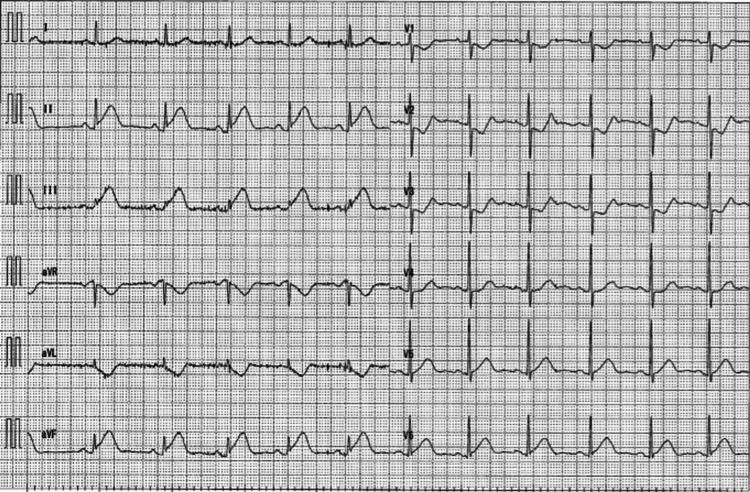
Electrocardiogram Electrocardiogram shows ST elevation on the Ⅱ, Ⅲ, and aVf leads

**Figure 3:**
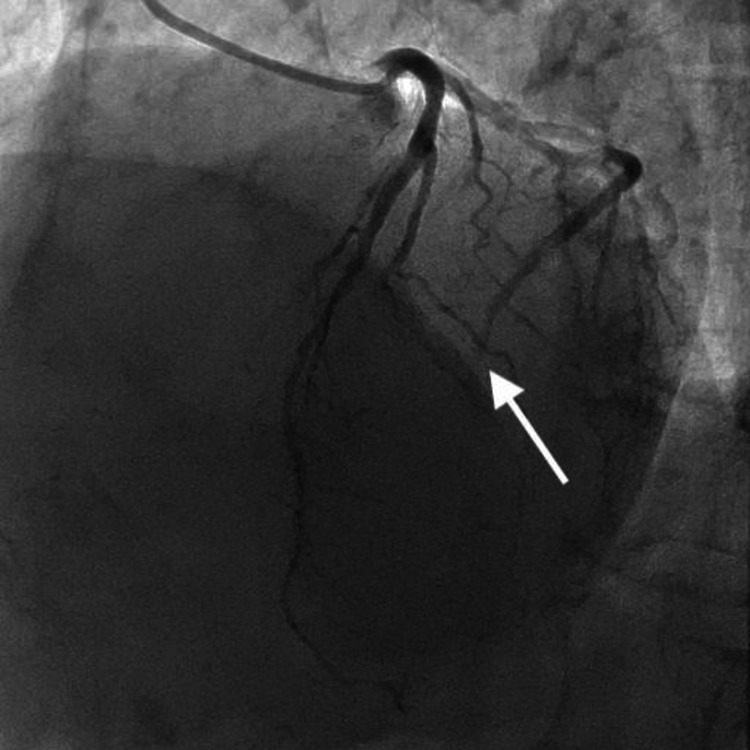
Coronary angiography image Coronary angiogram on admission in Case 1 shows the total occlusion of the mid circumflex

A 76-year-old woman was brought to our hospital by ambulance due to transient loss of consciousness (T-LOC) and hemi paralysis. The neurologic finding was improved on the emergency outpatient service though brain magnetic resonance imaging (MRI) showed new infarct findings in the upper right cortex (Figure 1), whereas an electrocardiogram showed ST elevation on the II, III, and aVf leads and the serum levels of cardiac enzymes were elevated (Figure 2). D-dimer at admission was 0.67 μg/ml (Table 1). An echocardiogram showed no focal wall motion abnormality or left ventricular thrombus and the ejection fraction was 78%. Then, an emergency coronary angiography and subsequently catheter aspiration was performed for the total occlusion of mid circumflex (Figure 3). She was admitted to an intensive care unit and was treated with continuous infusion of heparin 15,000 units/day in addition to the administration of bisoprolol fumarate 0.625 mg/day. A low dose of beta-blocker was started because she has no history of hypertension, and angiotensin-converting enzyme-inhibitor(ACE-I) was started at the same time. She was diagnosed with multiple thromboembolisms associated with asymptomatic and paroxysmal atrial fibrillation, which was evident from the electrocardiogram monitor on the third day. A continuous infusion of heparin was switched to edoxaban 30 mg/day on the fifth day. A low dose of edoxaban was started because her body weight was under 60 kg. And bisoprolol fumarate was increased to 1.25 mg/day on the same day. No thrombi were found on the other organ by contrast-enhanced computed tomography (CECT) examination on the ninth day, and coronary angiography confirmed the disappearance of the thrombus in the mid circumflex on the 20th day. Her general condition was stable, and no bleeding and extension of infarction was seen by head CT on the 21st day, thus she was discharged from the hospital.

**Table 1:** The parameters of transthoracic echo and D-dimer EF: ejection fraction; LVDD: left ventricular diastolic dysfunction; LAD: left anterior descending artery

Case	EF (%)	LVDD (mm)	LAD (mm)	MR	D-dimer (μg/mL)
1	78	40	36	trivial	0.67
2	71	31	38	mild	9.4

Case 2

**Figure 4:**
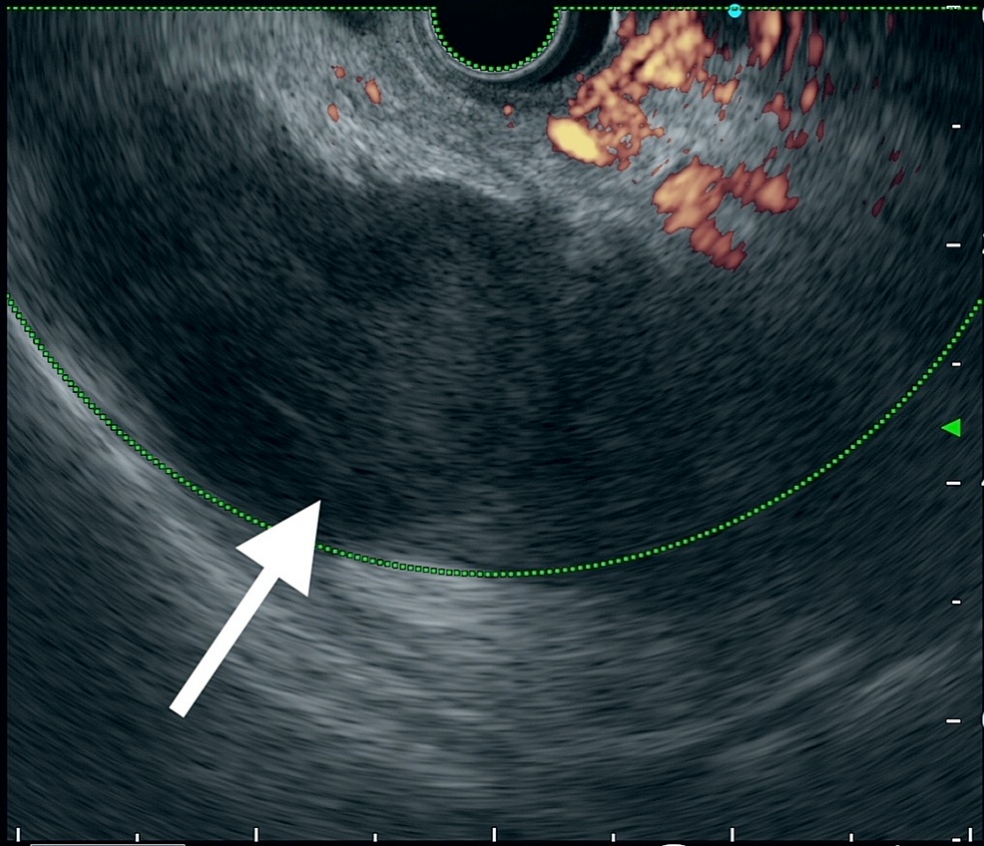
Endoscopic ultrasonography image Endoscopic ultrasonography on three days from admission in Case 2 shows the splenic infarction

**Figure 5:**
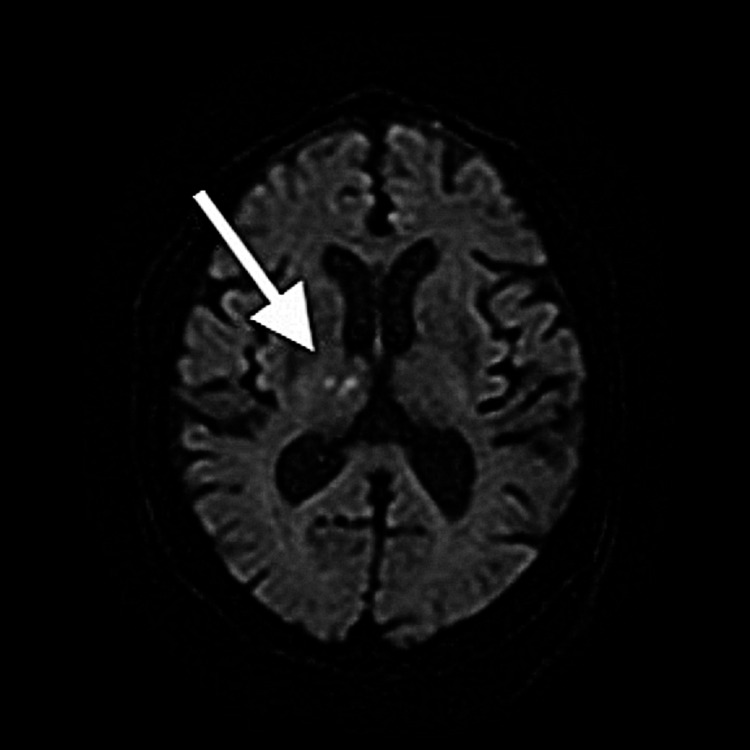
Brain MRI image Brain MRI on six days from admission in Case 2 shows infarctions in the right thalamus

An 88-year-old woman was admitted to ambulatory care for abdominal pain that lasted three days with no chest symptoms. An abdominal ultrasound showed a splenic infarction and atrial fibrillation was found by an electrocardiogram though it has not been detected by the medical examination for three years. Then, she was admitted to the hospital for the investigation of the association between splenic infarction and asymptomatic atrial fibrillation and was treated with continuous infusion of heparin 10,000 units/day. We did not know if she needed surgical resection for it, therefore we observed the use of heparin and switched to direct oral anticoagulant (DOAC) after conservative therapy. D-dimer at admission was 9.4 μg/ml (Table 1). An echocardiogram showed no thrombus in the left atrial and ventricular cavities and ejection fraction was 71% and the administration of verapamil 5 mg/day and bisoprolol fumarate 0.625 mg/day was started for the rate control. A low dose of beta-blocker was started because she had relatively low blood pressure and chronic kidney disease (CKD). A detailed investigation by endoscopic ultrasonography on the third day and brain MRI on the sixth day showed a splenic infarction (Figure 4) and multiple infarctions in the right thalamus and the left cerebellum (Figure 5). Thus, she was diagnosed with multiple thromboembolisms associated with asymptomatic AF. Then, a bisoprolol fumarate was gradually increased up to 7.5 mg/day for increased heart rate and continuous irregularity, and a continuous infusion of heparin was switched to apixaban 5 mg/day on the 14th day. Her general condition was stable, and no bleeding and extension of infarction were seen by a head and abdominal CT on the 20th day, thus she was discharged from the hospital.

## Discussion

We have shown two cases of multiple thromboembolic complications associated with undetected and asymptomatic AF, although thrombus was not found in the cardiac cavity or lower extremity vein. 

An undetected asymptomatic AF is considered to be a significantly high risk because the mortality per year is over two-fold higher in asymptomatic AF patients compared to symptomatic [5]. An asymptomatic and paroxysmal AF patient has less chance to receive an invasive intervention for rhythm control compared to a symptomatic AF patient, thus it raises concern about a progression to continuous AF [6], even if AF is found in an asymptomatic patient, anticoagulant therapy is not given in 69% of them for the concern about bleeding risk [4]. These findings indicated that an asymptomatic AF patient has a treatment-free interval using an antiarrhythmic agent and a risk of more frequent multiple thromboembolisms compared to symptomatic AF patients [7]. This complication sometimes contributes to fatal outcomes, thus the early detection of asymptomatic AF is very important.

The prediction and method for detecting AF include a CHA2DS2-VASc score of 2 points or higher, a HAS-BLED score of 2 points or higher [1], no history of oral β-blockers, and high NT-proBNP [4] were reported. This prediction is consistent with our cases; the CHA2DS2-VASc score was 4 points in Case 1 and 3 points in Case 2, whereas the HAS-BLED score was 1 point in Case 1 and 3 points in Case 2, and both cases did not have any history of β-blocker administration. This prediction method might be useful for undetected AF even though the NT-proBNP serum level was not measured in both cases.

It has been reported that the incidence of new cases of asymptomatic AF in the 70s is 3.7% per year, and it has been pointed out that AF may actually be more prevalent in the elderly [4]. Asymptomatic AF is difficult to detect because it has no subjective symptoms, and patients do not visit a medical institution and even ambulatory patients do not complain. The detection of AF in our cases was difficult even though the patient in Case 1 was visiting the clinic monthly for hypertension and the patient in Case 2 took an electrocardiogram during the annual medical checkups.

Regarding the detection rate of asymptomatic AF, it was significantly higher at 4.0% in 14-day external loop monitoring compared to 1.1% in the 24-hour Holter ECG [4], and it was 8.9% for a six-month observation, 12.4% for a 12-month observation, and 30.0% for a 36-month observation by an implantable cardiac monitor in patients with stroke or transient ischemic attack (TIA) within 90 days from onset who had no detection AF by ECG monitoring over 24 hours [8]. Also, it was 2.7% for a 12-lead ECG on admission, 4.1% for repeated ECG within five days, 5% for 24-hour Holter monitoring, and 5.7% for ambulatory ECG monitoring in stroke or TIA patients with no history of AF [9].

Those findings suggest that there is more existence of asymptomatic AF that were not detected by 24-hour Holter monitoring and the detection rate is expected to increase as the measurement time is extended.

Therefore, it is considered that self-checks by pulse rate and regulation at home may be useful for brief and non-invasive AF screening in elderly patients or high-risk patients with cardiac events. If there are any irregularities or a rapid pulse but it cannot be detected by Holter ECG, the doctor could consider the choice of a loop recorder test at a facility where can be inserted. The exact rate of undetected AF and thromboembolic events is unclear, and embolic complications are likely to increase as the rate of asymptomatic AF is probably high and will increase in the future.

## Conclusions

We reported two cases with multiple thromboembolic complications associated with asymptomatic AF.

For the future, simple self-examinations and prospective observational studies may clarify the presence of asymptomatic AF and the risk of embolism. So, a large clinical study is required to study the rate of undetected AF and thromboembolic events in asymptomatic patients.
